# ASBAAC: Automated Salt-Bridge and Aromatic-Aromatic Calculator

**DOI:** 10.6026/97320630014164

**Published:** 2018-04-30

**Authors:** Chittran Roy, Saumen Datta

**Affiliations:** 1 Structural Biology and Bioinformatics Division, Council of Scientific and Industrial Research - Indian Institute of Chemical Biology, 4 Raja SC Mullick Road, Jadavpur, Kolkata-700032, West Bengal, India

**Keywords:** salt-bridges, aromatic-aromatic contacts, protein, program, software

## Abstract

Biological systems are made of complex networks non-covalent interactions observed among protein-protein, protein-DNA, proteinlipid complexes using hydrogen-bonds, salt-bridges, aromatic-aromatic, van der Waals (vdW), hydrophobic-interactions and several others using distance criteria. Hence, large-scale data analysis is required to understand the principles of biological complex formation. Therefore, it is of interest to analyze non-covalent interaction namely, salt-bridge and aromatic-aromatic contacts in known and modeled protein complex structures. Here, we describe ASBAAC for automatic calculation of salt-bridges and aromatic-aromatic contacts in protein complexes. This software tool is fast, robust and user-friendly for large-scale analysis of inter-chain salt bridges and aromatic-aromatic contact in protein complexes.

**Availability:**

ASBAAC is available for free at http://sourceforge.net/projects/asbaac

## Background

Interaction between two or more proteins play a crucial role in maintaining cellular systems through non-covalent interactions such as hydrogen bonds, salt bridges and aromatic-aromatic (AA) contacts. A particular region where two or more proteins interact with each other is called the interface [1]. Knowledge of amino acids, which are involved in the formation of salt-bridges and A-A contacts with other interacting interfaces between various proteins, is important for the better understanding of protein-protein binding. Salt-bridges most often arise from the anionic amino acids (aspartic acid or glutamic acid) and the cationic amino acids (lysine, arginine or histidine) [2]. The interaction with these functional groups plays an important role in the formation of the structure and function of proteins. Formation of Salt Bridge between two residues occurs at a distance of 4Å [3]. There are numerous salt-bridge interaction strategies such as simple or complex, isolated or networked [4], intra- helical, coiled, strand or inter-helical, coiled and strand [5]. Mutagenesis studies and nuclear resonance technique reveal that the contribution of salt-bridge plays an important role in the overall stability of proteins.

Apart from salt-bridge, the aromatic residue interactions also play crucial role in protein stabilization, protein-protein recognition, ligand binding and protein folding [6]. On average 60% of aromatic side chains of protein involves in the formation of aromatic pair and 80% of which involves in the formation of a network of three or more interacting aromatic side chains [7]. Interaction occurs when pi rings range is between 4.5 to 7.5 Å assisting in the formation of pi-pi stacking [8].

There is about fifty thousand (until February 2018) highresolution (≤ 2.5 Å) multi-chain protein structure available at the protein data bank (PDB) used for the analysis of salt-bridges and aromatic-aromatic contacts. There has been extensive research on hydrogen bonds. However, data on salt-bridges and aromatic- aromatic interactions is limited. Therefore, there is a need to develop computer software tools to analyze S-B and A-A interaction of multi-chain proteins using known structure complexes. We describe ASBAAC for the large-scale analysis of S-B and A-A. ASBAAC is fast, robust, simple and user-friendly. ASBAAC is freely available for academic use.

## Program input

ASBAAC uses .pdb file format as input. This program scans the PDB text file and calculates the inter-chain salt bridge interaction and aromatic-aromatic contacts. ASBAAC should be run from UNIX prompt in directory containing PDB files for large-scale analysis.

## Program output

**Figure 1:**
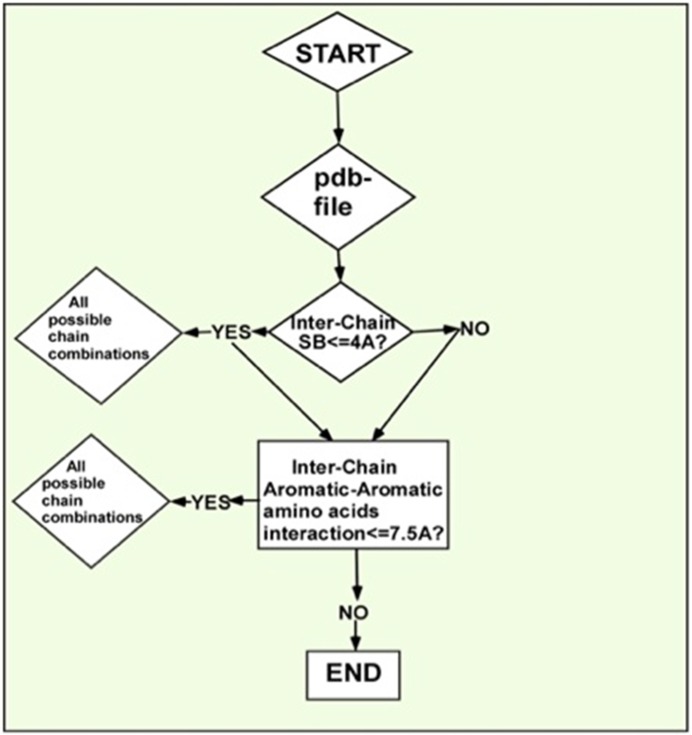
A flowchart showing the working procedure of ASBAAC. The first function is to scan the pdb file. If distance between atomic SB is within 4Å (i.e. YES), ASBAAC calculates all possible chain combination and goes to next step. If distance between atomic SB not within 4Å (i.e. NO), it comes out and goes to next level. Then the program enters the next lap to calculate pi-pi interaction. If the distance is within 7.5 Å (i.e. YES) it calculates all possible chain combinations until end of program.

Outputs are generated in the same working directory. A detail flow-chart for ASBAAC is shwon in Figure 1. It checks for chain continuity and then constructs a sequence while scanning the topology of the structure .pdb file. ASBAAC then calculates the distance between amino acids identifying salt-bridges, and aromatic-aromatic contacts in all possible chain combinations. The program runs at three modes. First mode checks the saltbridge interactions, second mode extracts aromatic interactions and third mode combines all possible results to produce output in text format within the same working directory.

## Caveats and future development

ASBAAC software is written in AWK programming language, which can be successfully run from C shell Unix prompt in 32-bit CYGWIN OS. It can also be made run from B shell LINUX and WINDOWS environment. We are developing web interface to integrate ASBAAC in future development.

## Methodology

### Distance calculation

Inter-atomic SB and A-A distance in inter-chain protein is calculated. Inter-atomic distance between charged amino acids of two chains is calculated using equation 1 given below.

$\mathrm{dist =}\sqrt{{\mathrm{(Xa-Xb)}}^{2}{\mathrm{+ (Ya-Yb)}}^{2}{\mathrm{+ (Za-Zb)}}^{2}}$

Here, a and b are specified side chain atoms of acidic and basic residues respectively. X, Y and Z are atomic coordinates.

This rule is also applicable for aromatic-aromatic interaction. In case of tyrosine, phenylalanine, histidine and tryptophane (along with its indole ring), the distance between any of these residues is calculated by taking the distance between the centroids of aromatic rings.

### Salt-bridge and aromatic-aromatic bond calculation

Positively charged atoms are Lys NZ1, Arg NH1, Arg NH2 and His NE2. Negatively charged atoms are Asp OD1, Asp OD2, Glu OE1, Glu OE2. A salt bridge is defined if two oppositely charged atoms lie within 4Å across the interface. A pi-pi interaction is defined between two aromatic amino acids if the distance calculated from their centroid is less than 7.5Å.

### Analysis of NMR solved structure

**Figure 2:**
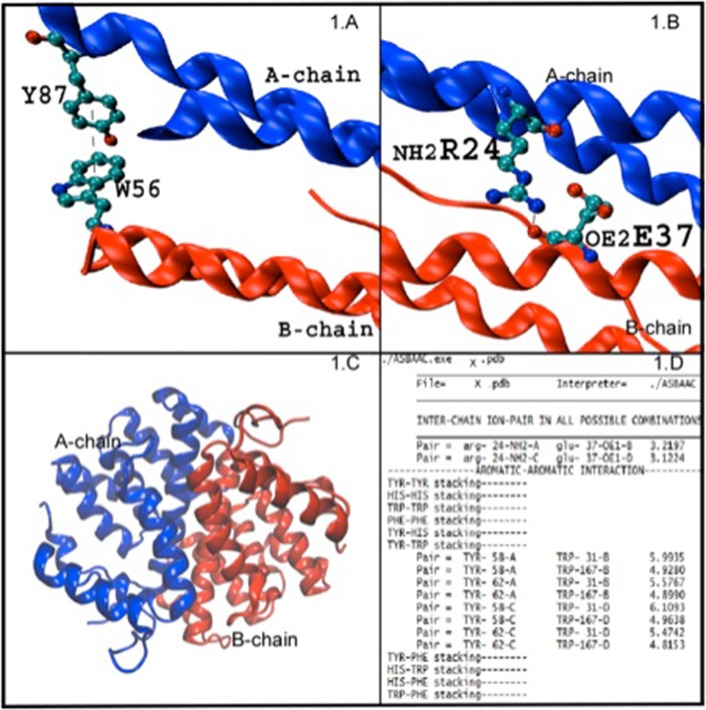
Details of ASBAAC extracted aromatic-aromatic (A) and salt-bridge (B) of multi-chain protein (C) analysis (D). X denotes any complex/multi-chain protein PDB ID.

ASBAAC analyzes inter-chain salt-bridge and aromatic-aromatic interactions at the interface of homo-meric or hetero-meric subunits. However, the program does not work for NMR structures with variable number of conformers containing homomeric or hetero-meric subunit structures. Analysis should be completed after separating the conformers in such cases (Figure 2).

## Abbreviations

S-B: Salt-Bridge
A-A: Aromatic-Aromatic
